# Case of Congenital Absence of One Testicle

**Published:** 1841-01-30

**Authors:** M. L. Anderson


### *Case of Congenital absence of one Testicle.*

To the Editors of the Medical Examiner.

*Gentlemen.*—In No. 30, of Vol. 3d of the Medical Examiner, I see the report of a case in which a testis was found in the abdomen of a man post mortem, by Geo. W. Bayless, M. D., in making an autopsical examination at the Louisville Marine Hospital, in May last. Dr. B., in his report, seems to infer or think that the descent of the testis was arrested by a mechanical cause; he also suggests the value this case may have upon legal medicine, and as connected with the question of contraction, which sometimes comes up in courts of justice. For the purpose of throwing further light on this subject, I am authorised to state the case of Mr. A.——, æt. 35 years, five feet ten inches high, weighs one hundred and sixty pounds, masculine and manly in appearance, and now in perfect health, had, at birth, but one testis in the scrotum, nor has he since had any increase in the number; the one he has appears natural in every respect. He was married at the age of twenty-six, his wife has one living healthy child, and has lost several others at an early period of pregnancy, owing, no doubt, to her general health being bad. Mr. A.'s grandfather (Mr. L.) was a captain in the revolutionary war, and the father of four children; his situation in regard to his testicles was the same in every respect as those of his grandson, Mr. A.

Respectfully, your obedient servant,

M. L. Anderson.

